# Effektivität, Effizienz und Sicherheit der Schlaganfall-Telemedizin in Zeiten der Corona-Pandemie: Der „Fall“ Thüringen

**DOI:** 10.1007/s00115-020-00970-5

**Published:** 2020-08-03

**Authors:** Caroline C. Klingner, Stefan Brodoehl, Franziska Wagner, Jörg Berrouschot, Albrecht Günther, Otto W. Witte, Carsten M. Klingner

**Affiliations:** 1 Hans Berger Klinik für Neurologie, Universitätsklinikum Jena, Friedrich-Schiller-Universität Jena, Erlanger Allee 101, 07747 Jena, Deutschland; 2 Biomagnetisches Zentrum, Universitätsklinikum Jena, Friedrich-Schiller-Universität Jena, Jena, Deutschland; 3 Klinik für Neurologie, Klinikum Altenburger Land GmbH, Altenburg, Deutschland

## Hintergrund

Weltweit zeigt sich in schwer von der Corona-/COVID-19-Pandemie betroffenen Regionen ein deutlicher Einbruch von Patientenvorstellungen mit akutem Schlaganfall oder einer transitorischen ischämischen Attacke (TIA; [1]). Dieser rückläufige Trend scheint auch telemedizinische Schlaganfallnetzwerke einzuschließen (bspw. Fallreduktion von bis zu 50 % in einer Region um Sevilla [2]) und ist insofern gefährlich, als dass die Vermeidung oder Verzögerung einer ärztlichen Vorstellung die erwähnte Patientengruppe weit mehr gefährdet als das COVID-19-Übertragungsrisiko im Krankenhaus. Ob sich in Deutschland, und hier insbesondere in von der COVID-19-Pandemie wenig betroffenen Regionen, ein ähnliches Bild zeigt, ist aktuell noch unklar. In diesem Artikel analysieren wir Daten des Schlaganfall-Telemedizin-Netzwerks in Thüringen (SATELIT) mit 20 angeschlossenen Kliniken.

## Methode

Es wurde eine retrospektive Analyse der Schlaganfallversorgung in Thüringen vor und nach dem COVID-19-Ausbruch für Patienten des Schlaganfallnetzwerkes in Thüringen (SATELIT) durchgeführt (20 Krankenhäuser). Erfasst wurden die Patientenzahlen aus dem Zeitraum 01.12.2019 bis 31.05.2020 sowie aus demselben Vergleichszeitraum des Vorjahres. Analysiert wurde die Anzahl aller Patienten, die im Rahmen von Telestroke-Konsultationen vorgestellt wurden, sowie insbesondere die Anzahl der Patienten mit den Diagnosen ischämischer Schlaganfall (ICD I63.-) und zerebrale transitorische ischämische Attacke (TIA, ICD G45.-). Gruppenvergleiche erfolgten mittels ungepaarter t‑Tests, wobei *p* < 0,05 als statistisch signifikant gewertet wurde.

## Ergebnis

**Figure 1:**
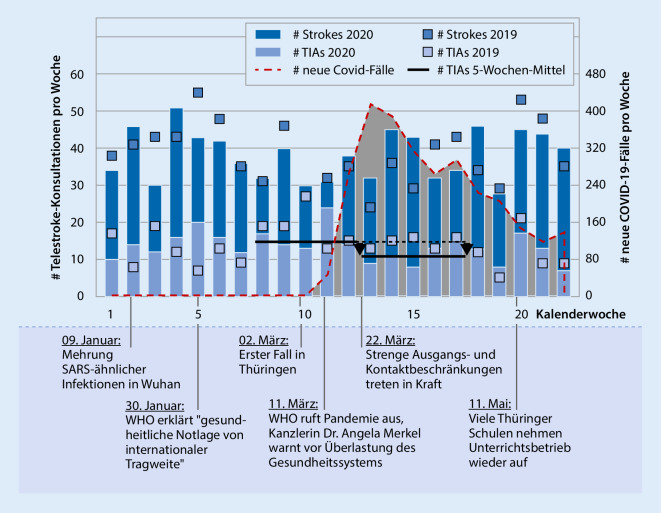

Die Anzahl der Patienten mit der Diagnose ischämischer Schlaganfall in der telekonsiliarischen Vorstellung in den 5 Wochen nach dem Erlass der Kontaktbeschränkungen (Lockdown) am 22.03.2020 im Vergleich zu den 5 Wochen davor stieg nicht signifikant von 174 auf 183 Fälle an (+5 %). Gegenüber demselben Zeitraum des Vorjahres (22.03.2019 bis 25.04.2019) zeigte sich ebenfalls keine signifikante Veränderung (165 Fälle vs. 183 Fälle; +9,8 %; Tab. 1; Abb. 1).

**Table 1:** 

		5 Wochen Prä-Lockdown vs. Lockdown		5 Wochen 2019 vs. Lockdown		Prä-COVID-19 vs. COVID (Erster COVID-19-Fall in Thüringen bis 31.05.2020)		Erster COVID-19-Fall in Thüringen bis 31.05.2020 vs. 2019	
Zeitraum		16.02–21.03.2020	22.03.–25.04.2020	22.03.–25.04.2019	22.03.–25.04.2020	01.12.2019–01.03.2020	02.03.–31.05.2020	02.03.–31.05.2019	02.03.–31.05.2020
Tage		35	35	35	35	90	90	90	90
TIA	*n*	85	53	73	53	205	165	185	165
	Fälle/Tag	2,8 ± 1,5	2,0 ± 1,0	2,7 ± 2,0	2,0 ± 1,0	2,6 ± 1,4	2,3 ± 1,3	2,5 ± 1,6	2,3 ± 1,3
	Veränderung	−37,6 %		−27,4 %		−19,5 %		−10,8	
	Cohens D	0,67		0,56		0,25		0,17	
	*p*-Wert	**0,015**		**0,046**		0,13		0,31	
Stroke	*n*	174	183	165	183	514	488	470	488
	Fälle/Tag	5,0 ± 2,3	5,4 ± 2,7	4,9 ± 2,3	5,4 ± 2,7	5,6 ± 2,6	5,4 ± 2,5	5,2 ± 2,3	5,4 ± 2,5
	Veränderung	+5 %		+10 %		−5,1 %		+3,7	
	Cohens D	0,16		0,21		0,09		0,11	
	*p*-Wert	0,50		0,39		0,55		0,47	
Lyse	*n*	21	15	26	15	57	50	66	50
	Fälle/Tag	0,6 ± 0,7	0,43 ± 0,6	0,74 ± 0,7	0,43 ± 0,6	0,63 ± 0,7	0,56 ± 0,8	0,73 ± 0,8	0,56 ± 0,8
	Veränderung	−29 %		−42 %		−12 %		−24 %	
	Cohens D	0,26		0,48		0,11		0,23	
	*p*-Wert	0,28		**0,049**		0,47		0,12

Die gestellte Diagnose TIA reduzierte sich statistisch signifikant in den 5 Wochen nach Beginn des Lockdowns von 82 auf 53 Fälle (−35,4 %). Diese Verminderung war im Vergleich zum Vorjahr ebenfalls signifikant (73 Fälle vs. 53 Fälle; −27,4 %, Tab. 1; Abb. 1). Die Anzahl der Lysetherapien sank um 28 % (21 vs. 15, *p* = 0,3) im Vergleich zu den 5 Wochen vor COVID-19 und um 42 % im Vergleich zum Vorjahreszeitraum (26 vs. 15, *p* = 0,049). Betrachtet man die gesamte Zeitspanne seit dem ersten Auftreten von Patienten in Thüringen, die positiv auf COVID-19 getestet wurden, bis Ende Mai (02.03.2020 bis 31.05.2020), so finden sich hier ähnliche Tendenzen. Jedoch erreichen diese dann über diesen längeren Zeitraum nicht mehr die statistische Signifikanz.

## Schlussfolgerung

Im Thüringer Telemedizinnetzwerk (SATELIT) zeigte sich in den 5 Wochen nach dem Lockdown eine Reduktion der TIA-Diagnosen und erfolgten Lysetherapien um ca. 30 %. Die Anzahl an Schlaganfalldiagnosen nahm hingegen nicht ab. Dies deutet auf eine Zurückhaltung von Patienten mit leichten Schlaganfallsymptomen hin, sich in den Kliniken vorzustellen, wie sie auch in schwer von COVID-19 betroffenen Regionen Europas oder den USA beobachtet wurde [3].

Begründet werden Rückgänge der Patientenzahlen einerseits mit Ängsten der Patienten vor einer nosokomialen Infektion, andererseits wird auch eine Überlastung des Gesundheitssystems mit schwer kranken COVID-19-Patienten in besonders schwer betroffenen Regionen angeführt, was vor allem zu einer geringeren Beachtung von Patienten mit nur leichten Schlaganfallsymptomen führt [1]. In Thüringen gab es zu keinem Zeitpunkt eine Knappheit in der Versorgung von Schlaganfallpatienten. Entsprechend ist hier die Angst der Patienten vor nosokomialen Infektionen als wesentliche Ursache der Fallzahlreduktion festzustellen. Eine Verzögerung des Notrufs durch eine solche Angst kann auch die geringere Lyserate erklären. Der fehlende Rückgang an Schlaganfalldiagnosen (sogar mit einer Tendenz zu mehr Schlaganfällen) könnte auf einer Konversion von nicht vorstellig werdenden TIA-Patienten zu Schlaganfallpatienten hindeuten.

Die hier beschriebenen Beobachtungen zeigen, dass die telemedizinische Versorgung von Schlaganfallpatienten auch in der pandemischen Lage kaum beeinträchtigt wurde. Der Zugang zu einem neurologischen Facharzt ist mittels Telemedizin einfach, schnell und vor allem ohne ein erhöhtes COVID-19-Übertragungsrisiko möglich.

Zusammenfassend zeigen die Daten, dass Ängste vor einer ärztlichen Vorstellung auch unabhängig von der tatsächlichen Gefahr in einer Region einen Einfluss auf die Vorstellung der Patienten in der Klinik haben können. Im Falle einer Vorstellung des Patienten ist jedoch die telemedizinische Versorgung der Patienten auch in Zeiten der Corona-Pandemie effektiv, effizient und hygienisch sicher. Wir halten daher die Telemedizin für einen wichtigen Bereich der Medizin, der nicht nur im Hinblick auf die aktuelle COVID-19-Pandemie einen wichtigen Stellenwert einnimmt.
